# Early evidence of flavored tobacco product restrictions in Massachusetts and New York State

**DOI:** 10.18332/tid/172000

**Published:** 2023-10-24

**Authors:** Barbara Schillo, Elizabeth L. Seaman, Alison Cuccia, Fatma Romeh M. Ali, Jamie Cordova, Sarah Mills, Jennifer Kreslake

**Affiliations:** 1 Truth Initiative, Washington, United States; 2 CDC Foundation, Atlanta, United States; 3 Department of Prevention and Community Health, Milken Institute School of Public Health, The George Washington University, Washington, United States; 4 Department of Health, Behavior, and Society, Bloomberg School of Public Health, Johns Hopkins University, Baltimore, United States

**Keywords:** policy impact, sales, Massachusetts, New York, e-cigarette policy

## Abstract

**INTRODUCTION:**

With many US states and localities enacting policies that restrict flavored e-cigarette sales, evaluation of these restrictions is critical to inform future efforts. This study analyzed both survey and retail scanner data to assess early-stage impacts of flavored tobacco sales restrictions in Massachusetts and New York State on e-cigarettes sales and product use among young people.

**METHODS:**

This study uses state-level e-cigarette retail sales data and survey data from youth and young adults (aged 13–24 years). Cross-sectional surveys were conducted at two time points in Massachusetts (both post policy implementation) and New York (pre and post policy implementation); retail sales data in both states were analyzed from 2019 through 2020 and compared to sales in control states.

**RESULTS:**

E-cigarette unit sales decreased significantly following the implementation of statewide restrictions on flavored e-cigarettes in both Massachusetts and New York State (p<0.001). Survey data showed a decrease in mint flavored e-cigarette use in Massachusetts and an increase in tobacco flavored e-cigarette use in New York State over time (p=0.001). In both states, a greater proportion of respondents reported using disposable e-cigarettes at Time 2 compared to Time 1 (p=0.001). Among those who reported using fruit-flavored e-cigarettes in New York State, a significantly greater proportion reported disposable device use at Time 2 compared to Time 1 (p=0.004).

**CONCLUSIONS:**

Findings from these case studies from two US states suggest that statewide policies reduce the availability of e-cigarettes and have the potential to reduce use of many youth-appealing flavors. The increase in use of disposable e-cigarettes likely reflects existing loopholes in federal policy, which may be attenuating the potential impact of strong state-level policies.

## INTRODUCTION

There is strong evidence that flavors attract young people to tobacco and nicotine products. For example, two-thirds of youth who use tobacco products reported flavors^1^ as a reason for use, and in 2020, and more than 85% of high school students who use tobacco had used a flavored product in the past 30-days. Most young people report that the first tobacco product they used was flavored^2^ and our most recent national studies continue to show that most youth reporting use of e-cigarettes report using flavored products^3,4^.

E-cigarettes first entered the US marketplace in 2007, and by 2017, e-cigarettes were available in more than 15500 unique flavors^5^. E-cigarettes quickly became the most commonly used tobacco product among young people with 14.1% of high school students and 3.3% of middle school students reporting past 30-day e-cigarette use in 2022. Intensity of use is a concern, 46.0% of high school and 20.8% of middle school students who vaped reporting using e-cigarettes 20 or more times in the past thirty days. Further, 85.5% of high school students and 81.5% of middle school students who use e-cigarettes had used a flavored e-cigarette product^3^, with the most common products flavored like fruit, mint, and candy, highlighting the important role flavors have in youth e-cigarette use.

Given nicotine’s unique effects on the adolescent brain including propensity for future drug abuse, dysfunction of emotional regulation, and cognitive deficits, as well as increased likelihood of continued and future tobacco use, use of e-cigarettes among young people is a serious public health concern^6,7^. The initial growth in youth e-cigarette use has largely been attributed to the product JUUL, a device with flavors such as mango and mint which are popular among high school students and the first e-cigarette known to deliver nicotine salts, which allow for high levels of nicotine to be consumed with less harshness than freebase nicotine^8^. In response to a growing outcry for the Food and Drug Administration (FDA) efforts to limit flavors in e-cigarettes, JUUL voluntarily removed all fruit and dessert flavored products from online and retail markets by November of 2018, followed by the removal of all mint flavored products in October of 2019^9^. However, the market quickly evolved to meet the consumer demand for flavored products, and sales of menthol JUUL pods grew^10^, along with sales of fruit flavors from other e-cigarette brands^11^.

Federal action, restricting flavored tobacco products to prevent youth use, contains exemptions that threaten public health. The FDA issued national guidance that prohibits the sale of flavored refillable e-cigarette cartridges other than tobacco or menthol in 2020. However, this policy excluded disposable e-cigarettes or e-liquids, which has led to a significant increase in sales of flavored disposable e-cigarettes.

In the absence of comprehensive federal oversight of flavored tobacco products and of flavored e-cigarettes in particular, state and local jurisdictions began to restrict the sales of all flavored tobacco products to address the growing e-cigarette epidemic^12,13^. As of 31 March 2023, 388 local and state policies restrict the sale of flavored tobacco products^13^.

In November 2019, Massachusetts became the first state to pass a law prohibiting the sale of all flavored tobacco products, including flavored e-cigarettes, menthol cigarettes and flavored cigars, except for on-site consumption at smoking bars, a class of licensed establishments with enclosed indoor spaces such as cigar bars or hookah bars^14,15^. The law went into effect immediately for flavored e-cigarettes, and on 1 June 2020 for all other flavored tobacco products^15^. Subsequently, New York State passed a statewide ban on flavored e-cigarettes that went into effect on 18 May 2020^16^. While the Massachusetts and New York State policies are similar, they differ in the scope of their product bans; Massachusetts bans all flavored tobacco products whereas New York State prohibits the sale of flavored e-cigarettes, but not other flavored products like little cigars, cigarillos and menthol cigarettes, and exempts flavored products that have an FDA pre-market approval^17^.

Although the evidence is still emerging on the impact of these policies, a recent systematic review of the published studies found that flavored and menthol tobacco product sales restrictions implemented in US jurisdictions have achieved some of their intended outcomes. The evidence was strongest for proximal impacts of reduced access and reduced consumption (as measured by proxy with retail sales data) of flavored tobacco products^12^. Several recent studies have assessed the impact of the state and local flavor policies across the US^18-22^. Compliance with such policies appears to be high with fewer flavored products available in stores^19,21,22^ assessed with retail sales data, environmental scans and research with retailers^23^. Survey data findings are mixed; even with strong policies and compliance, youth who use e-cigarettes may still be able to access flavored products in California^18^ and ever and current use has not changed^24^. However, work coming out of Minnesota indicates that youth use of flavored products may be stalled in areas with policies, compared to increasing in areas without policies^20^. Despite recent research, additional evidence is needed to fully understand the short- and long-term impacts of state and local flavored e-cigarette policies. Using both retail sales data and online survey data to assess the impact of flavor policies, we examined product sales and tobacco use behavior including use of flavors in two states. We also examined device type over time. While neither statewide policy explicitly regulated device type, the 2020 FDA guidance included differential regulations for device types, and prior studies have found certain device types are more likely to be sold in flavors other than tobacco^25^.

## METHODS

### Data sources

*Retail sales data*

Data were purchased from Information Resources, Inc. (IRI) and were aggregated by states in 4-week periods from 27 January 2019 to 27 December 2020. E-cigarette unit sales and product characteristics are captured at the point of sale from the Universal Product Code (UPC), a bar code designation for consumer products. Data provide state-representative sales from brick-and-mortar convenience stores, gas stations, grocery stores, drug stores, club stores, discount/dollar stores, mass merchandisers, and military commissaries. Data do not include sales from tobacco stores, vape shops and online retailers.

*Web survey data*

Repeated, cross-sectional online surveys were conducted at two time points. The survey used convenience sampling of respondents aged 13–24 years living in Massachusetts and New York State. Sampling quotas for age and gender were employed and post-stratification weights using Census demographic benchmarks were applied to resemble the population characteristics in each geographical area. This approach is commonly used for convenience sample internet panel surveys^26^. Invitations were sent through survey vendors to participants enrolled in Ipsos Public Affairs online survey panels and verified by home zip code. The survey study protocol was approved by Advarra Institutional Review Board (Protocol 00041490) on 23 January 2020. Parents of youth participants (aged 13–17 years) provided informed consent and the youth provided assent to participate. Young adults (aged 18–24 years) provided informed consent to participate. Participants received an incentive for completing the survey, generally between $10 and $15 with participants living in a house without internet access and participants from underrepresented backgrounds receiving higher incentives.

**Figure 1:**
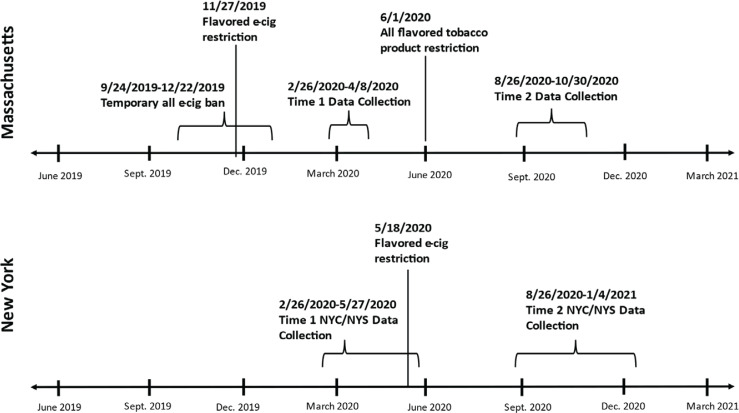
Timeline of policy implementation and web survey data collection and in Massachusetts and New York State, 2019–2021

Survey fielding dates relative to policy implementation can be found in Figure 1. In Massachusetts, both surveys were conducted after policy implementation of the permanent statewide e-cigarette flavor policy; the Time 1 survey was administered three months post policy (n=1042) and the Time 2 survey nine months post policy (n=990). Respondents in the New York City sample were combined with respondents from a New York rest-of-state sample and weighted into a single statewide dataset (n=2605 at Time 1, and n=2731 at Time 2). Over 90% of respondents in the Time 1 survey took the survey prior to the implementation of the state ban on flavored e-cigarettes (excluding tobacco) (n=1336; 93.4%); the Time 2 survey was administered 3–5 months post policy implementation.

### Measures

*Retail sales data*

E-cigarette flavors were categorized as tobacco, menthol, mint, all other flavors, and not available/applicable based on explicit flavor names^25,27^. E-cigarette products were classified as tobacco-flavored if tobacco or a descriptor (e.g. traditional, original, classic) was explicitly mentioned in the name of the product’s flavor. Flavors with a cooling sensation, such as ‘frost menthol’, were classified as menthol. Mint-flavored e-cigarettes, such as ‘wintergreen’, were classified as mint. The all-other flavor category included flavors such as fruit, clove/spice, candy/desserts/other sweets, chocolate, alcoholic drinks, and non-alcoholic drinks. Flavors that could not be readily identified (e.g. ‘Fusion’) were searched online using brands and product characteristics, then assigned to tobacco, menthol, mint, or all other flavors, based on their descriptions if possible.

Furthermore, all e-cigarette products were categorized as prefilled cartridges, disposable devices, or e-liquids. Tanks, cartridges, and pods used in rechargeable and reusable e-cigarette devices were classified as prefilled cartridges. Devices that are not intended to be reused or refilled were classified as disposable devices. E-liquids reflect those products which are containers of the liquid used in e-cigarette devices. Devices and accessories sold without e-liquids were excluded.

*Web survey data*

Characteristics

Demographic characteristics including age (13–17, 18–20, 21–24 years), race and ethnicity (White Non-Hispanic, Black Non-Hispanic, Other Race Non-Hispanic, and Hispanic), gender (male, female) and perceived financial situation (live comfortably, meet needs with a little left, just meet basic expenses, don’t meet basic expenses) were assessed.

Flavor use

Respondents who had used an e-cigarette in the past 30-days (current) were asked to select all flavors they used in the past 30-days. Current flavors used were categorized into categories of tobacco, menthol, mint, fruit, and other (clove or spice, chocolate, alcoholic drink, candy dessert or other sweet, and other). Since these flavor categories were ‘select all that apply’, respondents could use multiple flavor categories.

Device type

Respondents who had used an e-cigarette in the past 30-days (current) reported the device type of e-cigarette used most often in the past 30-days: a disposable e-cigarette, an e-cigarette that uses pre-filled or refillable pods or cartridges (pod mods), or an e-cigarette with a tank that can be refilled with liquids (tanks/mods).

### Statistical analysis

*Retail sales data*

**Figure 2:**
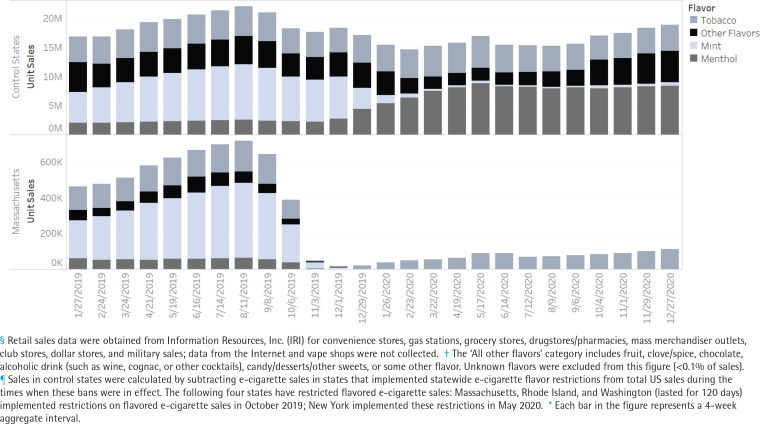
E-cigarette unit retail sales^§^, by flavor^†^, in control states^¶^ versus Massachusetts, 2019–2020*

**Figure 3:**
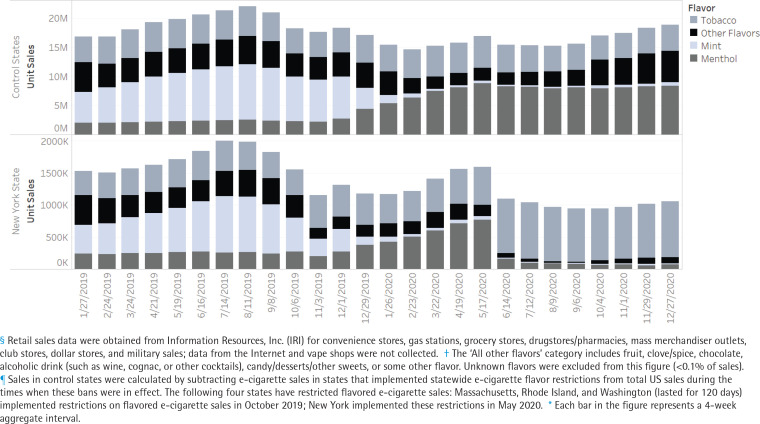
E-cigarette unit retail sales^§^, by flavor^†^, in New York State versus control states^¶^, 2019–2020*

Trends in e-cigarette sales in Massachusetts and New York State from January 2019 to December 2020 were assessed overall, by flavor, and product type relative to sales in control states before and after policy implementation (Figures 2 and 3). Sales in control states were calculated by subtracting e-cigarette sales in states that implemented statewide e-cigarette flavor restrictions from total US sales during the times when these bans were in effect. The following four states have restricted flavored e-cigarette sales: Massachusetts, Rhode Island, and Washington (lasted for 120 days) implemented restrictions on flavored e-cigarette sales in October 2019; New York State implemented these restrictions in May 2020^28^. Additionally, some localities within states have restricted flavored e-cigarette sales which may contaminate the control group and bias the results. Timing of policy implementation, however, varied across localities within states which complicates the ability to exclude data at the state-level, based on a common date. As a sensitivity analysis, sales data in states with local flavor restrictions were excluded during 2020, given that most of the restrictions on flavored e-cigarette sales have begun in 2020. These states include California, Colorado, Georgia, Illinois, Maine, Ohio, and Oregon.

The main outcome was standardized unit sales, where one unit reflects 5 prefilled cartridges or 1 disposable device or 1 e-liquid bottle^28^. Due to low unit sales, e-liquid sales were not stratified and compared pre and post policy. However, these e-liquids were included in the calculation of total unit sales. Percentage changes along with 95% Confidence Intervals (CIs) were calculated. Regression analyses were used to assess the significance of changes in sales within Massachusetts and New York State as well as differences between Massachusetts and New York State and control states. A simple regression analysis with a pre-post indictor was used to assess the significance of changes in sales over time within each intervention state. A p-value of the indicator variable was reported for each state. To compare each intervention state with control states, a simple regression analysis with a pre-post indicator, a control-intervention indicator, and an interaction term between these two indicator variables was conducted. The p-value of the interaction term was reported. Sales data analyses were conducted in Stata 17 with findings significant at p<0.05.

*Respondent data*

Data were weighted using US Census estimates to reflect the demographic profile of each state’s population. Analyses were conducted in Stata SE 15.1 (College Station, TX). Pearson chi-squared tests were used to assess differences in e-cigarette use, flavors used, and device type between Time 1 and Time 2, in each state. All variables were reported and analyzed as frequencies. Differences in device type by flavor category were also examined from Time 1 to Time 2.

## RESULTS

### Massachusetts

*Sales data*

E-cigarette unit sales in Massachusetts decreased significantly following the implementation of statewide restrictions on flavored e-cigarettes in October 2019, a change that was immediate and sustained over time (Table 1). In particular, the average 4-week period total e-cigarette unit sales decreased by 89.4% (95% CI: -92.7 – -84.7) from October 2019 to December 2020, compared to the period before implementation of flavor restrictions (January to October 2019) (Table 1). This decline was significantly (p<0.05) greater than the decline in sales in control states during this period (-14.6%; 95% CI: -20.5 – -8.1). The decline in total e-cigarette sales in Massachusetts was statistically significant and consistent across all flavors and device types. Tobacco-flavored e-cigarette sales, however, experienced a smaller decline (-65.56%; 95% CI: -80.95 – -37.76) compared to non-tobacco flavored e-cigarettes (-99.8% to -100.0%; p<0.05).

**Table 1:** Changes in average 4-week period e-cigarette unit retail sale^a^ in Massachusetts and New York State following flavored e-cigarette restrictions^b^, relative to control states, 2019–2020

*Sales*	*Massachusetts (MA) vs control states^e^*			*New York State (NY) vs control states*		
	*Massachusetts*	*Control states*	*p (MA vs control states)*	*New York*	*Control states*	*p (NY vs control states)*
	*% Change (95% CI)^f^*	*% Change (95% CI)*		*% Change (95% CI)*	*% Change (95% CI)*	
**Total sales**	-89.42 (-92.7 – -84.67)	-14.55 (-20.52 – -8.13)	<0.001	-33.88 (-42.1 – -24.51)	-7.17 (-15.89– 2.44)	<0.001
**Sales by flavor**						
Tobacco	-65.56 (-80.95 – -37.76)	-3.67 (-9.53–2.57)	<0.001	83.42 (67.42–100.95)	-7.94 (-13.18 – -2.38)	<0.001
Menthol	-99.79 (-99.93 – -99.37)	185.5 (115.5–278.24)	<0.001	-74.64 (-81.97 – -64.34)	149.97 (68.41–271.03)	<0.001
Mint	-100.00 (-100 – -99.97)	-90.75 (-95.6 – -80.55)	<0.001	-94.47 (-97.72 – -86.62)	-89.13 (-95.69 – -72.58)	0.281
Other flavors	-99.91 (-99.99 – -99.28)	-26.76 (-42.02 – -7.48)	<0.001	-81.09 (-87.12 – -72.26)	-8.34 (-30.73–21.28)	<0.001
**Sales by product**						
Disposables^c^	-84.75 (-91.81 – -71.61)	73.20 (34.11–123.69)	<0.001	-26.32 (-46.25–1.01)	104.39 (66.7–150.57)	<0.001
Prefilled cartridges^d^	-89.80 (-92.91 – -85.32)	-26.28 (-32.47 – -19.52)	<0.001	-34.12 (-43.95 – -22.57)	-23.72 (-32.19 – -14.18)	0.137

In contrast to Massachusetts, trends in control states varied by flavor and device type during this period when national policy allowed for the sale of flavored disposable cigarettes. Menthol flavored e-cigarette sales in control states increased significantly (185.5%; 95% CI: 115.5–278.24), while sales of mint and all other flavored e-cigarettes decreased significantly (-90.8% and -26.8%, respectively). There was no significant change in tobacco-flavored sales. By product type, prefilled cartridge sales in control states decreased significantly (-26.3%; 95% CI: -32.5 – -19.5), while sales of disposal e-cigarettes (which are still available in all flavors) increased significantly during this period (73.2%; 95% CI: 34.1–123.7). This compares to over an 80% decrease in both disposable and prefilled cartridge sales in Massachusetts, where all flavored products were restricted.

*Survey findings*

Survey respondent characteristics across Time 1 and Time 2 are presented in Table 2. No significant differences related to respondent demographic characteristics were observed across the two samples. Massachusetts respondents at both Time 1 and 2 were largely non-Hispanic White and reported their financial situation as ‘living comfortably’. The samples were balanced on gender with about 40% of the samples comprising youth aged 13–17 years.

**Table 2:** Characteristics of e-cigarette product use reported by a cross-sectional sample of youth and young adults in Massachusetts and New York State over two survey periods, 2020–2021 (N=7368)

*Characteristics*	*Massachusetts*			*New York State*		
	*Time 1 (26 February to 8 April 2020) n (wt. %)*	*Time 2 (26 August to 20 October 2020) n (wt. %)*	*p*	*Time 1 (26 February to 27 May 2020) n (wt. %)*	*Time 2 (26 August 2020 to 4 January 2021) n (wt. %)*	*p*
**Total**	1042	990		2605	2731	
**Sociodemographic characteristics**						
**Age (years)**			0.985			0.987
13–17	352 (39.0)	385 (38.7)		1014 (39.8)	1127 (39.8)	
18–20	360 (27.5)	268 (27.3)		714 (26.4)	750 (26.1)	
21–24	330 (33.5)	337 (34.0)		877 (33.9)	854 (34.1)	
**Race/ethnicity**			0.998			0.995
White NH	676 (64.8)	671 (64.9)		1492 (50.3)	1476 (50.2)	
Black NH	72 (8.3)	59 (8.2)		319 (15.3)	363 (15.6)	
Other NH, 2+ races	129 (11.4)	118 (11.6)		318 (12.0)	349 (12.0)	
Hispanic	163 (15.5)	138 (15.2)		472 (22.5)	523 (22.3)	
**Gender**			0.825			0.778
Male	374 (50.0)	512 (50.6)		1196 (50.3)	1409 (50.8)	
Female	668 (50.0)	478 (49.4)		1409 (49.7)	1322 (49.2)	
**Financial situation**			0.155			0.866
Live comfortably	461 (48.6)	474 (50.6)		1264 (46.7)	1321 (47.5)	
Meet needs with a little left	350 (31.3)	321 (31.0)		850 (32.9)	872 (31.4)	
Just meet basic expenses	197 (16.6)	146 (13.1)		405 (15.8)	427 (16.3)	
Don’t meet basic expenses	29 (3.5)	47 (5.3)		80 (4.6)	103 (4.8)	
**E-cigarette use**						
Ever	462 (42.3)	399 (39.6)	0.317	1130 (38.9)	1189 (40.6)	0.329
Current	238 (22.7)	194 (19.3)	0.121	642 (20.1)	733 (22.2)	0.144
**E-cigarette characteristics among current users (past 30-days)**						
**Flavors used in past 30-days**						
Tobacco	55 (28.7)	59 (32.4)	0.523	121 (16.6)	213 (26.2)	0.001
Menthol	66 (30.5)	39 (21.1)	0.084	166 (26.4)	202 (27.4)	0.751
Mint	100 (40.8)	37 (20.7)	0.001	223 (36.7)	200 (30.2)	0.063
Fruit	114 (44.2)	89 (50.8)	0.265	308 (50.4)	318 (47.1)	0.363
Other	60 (25.0)	58 (32.0)	0.193	223 (32.4)	260 (35.9)	0.305
**Device type**			0.104			0.001
Disposable	71 (29.1)	70 (38.2)		180 (27.1)	260 (38.2)	
Pod mod	131 (56.0)	81 (43.0)		333 (48.7)	330 (45.6)	
Tank/mod	35 (14.9)	36 (18.8)		128 (24.2)	116 (16.2)	
**Flavors by device type**						
**Tobacco**			0.891			0.007
Disposable	15 (31.6)	20 (36.5)		32 (20.9)	76 (32.6)	
Pod mod	33 (53.2)	27 (47.6)		63 (46.1)	106 (53.7)	
Tank/mod	7 (15.2)	9 (15.9)		26 (33.1)	29 (13.7)	
**Menthol**			0.129			0.135
Disposable	14 (20.3)	15 (43.0)		32 (20.4)	71 (31.7)	
Pod mod	45 (68.5)	21 (50.2)		106 (55.4)	104 (53.0)	
Tank/mod	7 (11.3)	3 (6.9)		28 (24.1)	26 (15.3)	
**Mint**			0.934			0.64
Disposable	34 (34.9)	9 (34.0)		72 (32.5)	67 (38.3)	
Pod mod	61 (58.6)	23 (56.9)		114 (50.1)	90 (44.9)	
Tank/mod	5 (6.5)	4 (9.0)		37 (17.5)	36 (16.8)	
**Fruit**			0.062			0.004
Disposable	41 (33.3)	34 (41.0)		88 (30.2)	130 (45.5)	
Pod mod	53 (52.7)	28 (33.1)		148 (42.9)	125 (39.1)	
Tank/mod	20 (14.0)	25 (26.0)		72 (26.9)	52 (15.3)	
**Other flavors**			0.027			0.157
Disposable	16 (20.2)	24 (47.2)		59 (26.4)	85 (37.5)	
Pod mod	29 (58.0)	23 (39.2)		110 (48.8)	113 (40.9)	
Tank/mod	15 (21.8)	9 (13.6)		53 (24.8)	50 (21.5)	

Characteristics of e-cigarette product use are also presented in Table 2. Approximately 40% of youth and young adults in Massachusetts reported having ever used e-cigarettes, with approximately 20% reporting current use at both Time 1 and Time 2. Among respondents who used e-cigarettes in the past 30-days, significantly fewer Massachusetts respondents at Time 2 (20.7%) reported mint-flavored e-cigarette use than at Time 1 (40.8%, p=0.001) (Table 2). Significant differences were observed in device type among those who used other e-cigarette flavors; at Time 2, a greater proportion of respondents used disposable products (47.2%) and fewer respondents reported use of pod mods (39.2%) compared to Time 1 (disposable 20.2%, pod mod 58.0%, p=0.027).

### New York State

*Sales data*

E-cigarette unit sales in New York State decreased significantly following the implementation of a statewide ban on flavored nicotine e-cigarettes, excluding tobacco-flavored e-cigarettes. Specifically, the average 4-week period total e-cigarette unit sales decreased by 33.9% (95% CI: -42.1 – -24.5) from May to December 2020 compared to the period before implementation of flavored e-cigarette restrictions (January 2019 to May 2020) (Table 1). E-cigarette sales in control states did not significantly change during May to December 2020 compared to the period during January 2019 to May 2020.

New York State experienced a decline in total e-cigarette sales across all device types and flavors, with the exception of tobacco-flavored e-cigarettes. Notably, tobacco-flavored e-cigarette sales increased by 83.4% (95% CI: 67.4–101.0). Sales of tobacco flavored e-cigarettes decreased in control states during this period (-7.9%; 95% CI: -13.2 – -2.4). The significant decline in menthol e-cigarette sales in New York State (-74.6%; 95% CI: -82.0 – -64.3) stands in stark contrast to the increase in sales of menthol e-cigarettes in control states during the same period (150.0%; 95% CI: 68.4–271.0). Furthermore, while sales of disposal e-cigarettes increased significantly in control states during this period (104.4%; 95% CI: 66.7–150.6), both prefilled cartridge and disposal e-cigarette sales decreased in New York State.

*Sensitivity analyses*

Excluding states with local flavored e-cigarette restrictions from the control group did not meaningfully change the results. The direction and the significance of the trends were similar with some changes in magnitude. The results are reported in Supplementary file Table 1.

*Survey findings*

New York State survey respondent characteristics across Time 1 and Time 2 are presented in Table 2. No significant differences related to respondent demographic characteristics were observed across the two samples. Most New York State respondents, about 50%, were non-Hispanic White in both Time 1 and 2. Respondents were relatively evenly distributed by gender and about 40% of respondents were aged 13–17 years. Nearly half of the sample reported their financial situation as ‘living comfortably’ across the two samples.

Approximately one in five youth and young adults in our sample currently used e-cigarettes at Time 1. A similar proportion was observed at Time 2. Use of tobacco flavored e-cigarettes (26.2%) was significantly higher at Time 2 compared to Time 1 (16.6%, p=0.001). Respondents reported significantly higher use of disposable devices and lower use of tanks/mods at Time 2 (tank/mod: 16.2%, disposables: 38.2%) compared to Time 1 (tank/mod: 24.2%, disposables: 27.1%, p=0.001). Among young people who reported use of tobacco-flavored e-cigarettes in the past 30-days, significantly more reported using disposables (32.6%) and pod mods (53.7%) at Time 2 compared to Time 1 (disposables: 20.9%, pod mods: 46.1%, p=0.007). Additionally, among those who reported using fruit-flavored e-cigarettes, a significantly greater proportion reported disposable device use and lower use of pod mods at Time 2 (45.5%) than Time 1 (30.2%), (Time 2: 39.1%, Time 1: 42.9%) (p=0.004). Sample size, unweighted percentages and weighted percentages are included for both states in Supplementary file Table 1.

## DISCUSSION

In the months following the implementation of state policies to prohibit the sale of flavored e-cigarettes, both Massachusetts and New York State experienced significant declines in total e-cigarette unit sales in tracked channels. In Massachusetts, this decline was immediate, with a low level of total sales primarily consisting of tobacco flavored e-cigarettes. In New York State, tobacco flavored e-cigarettes sales increased, suggesting a consumer shift to those e-cigarettes that were still available in the market. These states stand in contrast to the national trends^29^ where e-cigarette sales remained high during the study period, with increases in sales in menthol e-cigarettes as well as disposable devices which are still available nationally in a wide range of flavors.

Survey data from Massachusetts indicate a significant reduction in the use of mint flavored e-cigarettes among youth and young adults. In New York State, youth and young people reported greater use of tobacco flavored e-cigarettes, the only type of e-cigarettes still legally available in New York. This finding is consistent with the sales data for that state. In Massachusetts, we see a slight decrease in use of menthol flavored e-cigarettes during a period in which national sales of menthol e-cigarettes dramatically increased.

These study findings are consistent with a recent study demonstrating high retailer compliance and large decreases in the sales of all flavored tobacco following policy implementation in Massachusetts^30^. Similarly, a study of the statewide flavor policy in New York found that while there were decreases in past 30-day e-cigarette use, cigarette, and dual use of e-cigarettes and cigarettes, were observed over the 2-year period in New York State post implementation; 95% of vapers still reported using a non-tobacco-flavored e-cigarette following the restriction^31^.

Findings provide insight into the immediacy of shifts in consumer behavior when flavor accessibility varies by device type. Both state policies were implemented during a period in which disposable e-cigarettes sales increased when they were exempted from the 2020 FDA Guidance which restricted flavored e-cigarettes. Data from the 2021 National Youth Tobacco Survey found that 53.7% of youth who currently use e-cigarettes reported the use of a disposable e-cigarette^26,32,33^. In this study, we see significantly higher use of disposables use among young people who currently use e-cigarettes in New York State over time; disposables use also increased in Massachusetts. These findings suggest that as long as the larger national market includes flavored e-cigarettes regardless of device type, youth remain at risk of using these products even in states with restrictions in place. It is possible that purchasing behavior also shifted to online or vape shops, but these retailer types are not provided in the retail sales data used for this study. Collectively, these behaviors support the need for comprehensive policies to further limit access to all nicotine and tobacco products that present harms to youth and young adults.

### Limitations

This study has several limitations. First, the retail sales data do not include demographic information of purchasers’ age. Thus, sales data cannot explain purchase patterns by age categories. However, prior research shows that current use of e-cigarettes among adults remains relatively low (4–5% nationally)^34^; the majority of those who use e-cigarettes continue to comprise youth and young adults^33^. Second, retail sales data only include in-store purchasing for a set of store types; these data cannot provide information about trends in online purchasing or vape/tobacco store purchasing. The potential access through these sources has implications for policy change; comprehensive efforts that include all types of retailers are needed. Third, survey samples were drawn using convenience sampling methods which, despite the use of sampling quotas and weights to approximate the population characteristics in each geographical area, limit the representativeness of the sample and thus the generalizability of the findings. Additionally, sales, behavior, and policy implementation in Massachusetts and New York State may not be generalizable to other states. Massachusetts had significant support at the local level prior to the implementation of a state-wide policy with 170 local flavor restrictions before a statewide ban was implemented. The statewide policies passed in New York State and Massachusetts also included other tobacco policy provisions that may have influenced sales and self-reported use patterns. Finally, the observational and cross-sectional nature of the survey cannot rule out that findings could be attributable to factors other than the policy changes, and additional research with stronger longitudinal designs will be needed to more fully attribute policy impacts.

Nonetheless, these results signal some early and important sales and behavior shifts in the period following policy implementation in two states. Findings are consistent with other published studies that show that state and local tobacco restrictions produce a dramatic and immediate impact on sales of e-cigarettes^12,19,28,35^. Prior studies have used retail sales indicators^19,21,22^ and survey data^18,20,24^ for early-stage policy evaluation. The present study builds on prior work by leveraging multiple data sources to create a more complete picture of flavored tobacco policy impacts.

### Implications

Future research is needed to examine the longer term behavioral impacts of these flavored tobacco policies and can leverage both ongoing surveys and health department surveillance efforts that measure state and local policy impacts. Research efforts should consider whether and to what extent state and local polices are implemented and enforced to more fully explain the impact of policy restrictions. Studies should also investigate unintended consequences of policy passage. While studies to date demonstrate that the Massachusetts policy was effective in reducing access to flavors without increasing sales in neighboring states^30,36^, this will be an important aspect of flavored tobacco policies to continue to monitor. Study findings reinforce the continued need to better understand access and patterns of use, and to address loopholes in the current regulatory environment at the federal level in order to maximize the impact of flavored tobacco sales restrictions.

## CONCLUSIONS

Findings from these case studies from two US states suggest that statewide policies reduce the availability of e-cigarettes and have the potential to reduce use of many youth-appealing flavors. The increase in use of disposable e-cigarettes likely reflects existing loopholes in federal policy which may be attenuating the potential impact of strong state-level policies.

## Data Availability

The data supporting this research cannot be made available for privacy or other reasons. The sales data used are available to purchase through IRi. The web survey data used are private.
