# Corrigendum: Early evidence of flavored tobacco product restrictions in Massachusetts and New York State

**DOI:** 10.18332/tid/175046

**Published:** 2023-11-11

**Authors:** Barbara Schillo, Elizabeth L. Seaman, Alison Cuccia, Fatma Romeh M. Ali, Jamie Cordova, Sarah Mills, Jennifer Kreslake

**Affiliations:** 1 Truth Initiative, Washington, United States; 2 CDC Foundation, Atlanta, United States; 3 Department of Prevention and Community Health, Milken Institute School of Public Health, The George Washington University, Washington, United States; 4 Department of Health, Behavior, and Society, Bloomberg School of Public Health, Johns Hopkins University, Baltimore, United States

**Keywords:** policy impact, sales, Massachusetts, New York, e-cigarette policy

Corrigendum on:

Early evidence of flavored tobacco product restrictions in Massachusetts and New York State

By Barbara Schillo, Elizabeth L. Seaman, Alison Cuccia, Fatma Romeh M. Ali, Jamie Cordova, Sarah Mills, Jennifer Kreslake

Tobacco Induced Diseases, Volume 21, Issue October, Page 1-14, Publish date: 24 October 2023

DOI: https://doi.org/10.18332/tid/172000

In the originally published version of the article, the name of the 4th author was given as Fatma R. M. Ali and is now changed to Fatma Romeh M. Ali. The mentioned change is corrected also online.

