# Supplementary material for: Early evidence of flavored tobacco product restrictions in Massachusetts and New York State
Source: Tob Induc Dis. 2023 Oct 24;21:140. doi: 10.18332/tid/172000 (PMC10594628; doi:10.18332/tid/172000)
Supplement: Supplementary file 1 [file TID-21-140-s1.pdf]

Appendix

Table A1: Changes in Average 4-Week Period E-Cigarette Unit Sales[1] in Massachusetts and New York Following Flavored E-Cigarette Restrictions[2], Relative to Control States, 2019-2020 (Excluding states with local flavor policies)

State	Massachusetts versus Control States[5]			New York versus Control States		
	Massachusetts % Change (95% CI)[6]	Control States % Change (95% CI)	P-value	New York % Change (95% CI)	Control States % Change (95% CI)	P-value
**Total Sales**	-89.42 (-92.7, -84.67)	-24.29 (-31.14, -16.76)	<0.001	-33.88 (-42.1, -24.51)	-16.74 (-27.54, -4.32)	0.017
**Sales by Flavor**						
**Tobacco**	-65.56 (-80.95, -37.76)	-16.44 (-22.35, -10.08)	<0.001	83.42 (67.42, 100.95)	-19.76 (-24.51, -14.7)	<0.001
**Menthol**	-99.79 (-99.93, -99.37)	153.42 (98.12, 224.15)	<0.001	-74.64 (-81.97, -64.34)	122.97 (56.84, 216.96)	<0.001
**Mint**	-100.00 (-100, -99.97)	-91.54 (-96.08, -81.75)	<0.001	-94.47 (-97.72, -86.62)	-89.82 (-96.12, -73.25)	0.34
**Other Flavors**	-99.91 (-99.99, -99.28)	-34.17 (-49.36, -14.42)	<0.001	-81.09 (-87.12, -72.26)	-15.51 (-38.98, 16.99)	<0.001
**Sales by Product**						
**Disposables[3]**	-84.75 (-91.81, -71.61)	52.43 (18.5, 96.07)	<0.001	-26.32 (-46.25, 1.01)	85.98 (53.38, 125.52)	<0.001
**Prefilled Cartridges[4]**	-89.80 (-92.91, -85.32)	-34.74 (-42.16, -26.37)	<0.001	-34.12 (-43.95, -22.57)	-32.11 (-42.13, -20.35)	0.785

*CI: Confidence Intervals

[1] Unit sales were standardized to reflect the most common package size by product type. A standardized unit was equal to: 5 prefilled cartridges; 1 disposable device; 1 e-liquid bottle.

[2] For Massachusetts, pre-intervention period is the period during Jan 2019-October 2019; post-intervention period is October 2019 – Dec 2020. For New York, pre-intervention period is the period during Jan 2019-May 2020; post-intervention period was May 2020– Dec 2020.

[3] *Prefilled cartridges include tanks, cartridges, and pods used in rechargeable and reusable e-cigarette device; the cartridges are not intended to be refilled after the liquid has been depleted.*

[4] *Disposable devices include nonrechargeable* and *nonreusable e-cigarette devices* that are *not intended* to be *refilled* with *e-liquid after being depleted*; the *device* is *disposed* of *once* the *e-liquid has been consumed.*

[5] Sales in control state were calculated by subtracting e-cigarette sales in states that implemented statewide e-cigarette flavor restrictions as well as states with local restrictions on flavored e-cigarette sales from total U.S. sales during the times when these bans were in effect. The following four states have restricted flavored e-cigarette sales: Massachusetts, Rhode Island, and Washington (lasted for 120 days) implemented restrictions on flavored e-cigarette sales in October

2019; New York implemented these restrictions in May 2020. Additionally, these states restricted flavored e-cigarette sales at the local level California, Colorado, Georgia, Illinois, Maine, Ohio, Oregon.

[6] Confidence intervals and P-values were calculated using t-tests that compare periods after versus periods before flavored e-cigarette restrictions as well as total U.S. versus Massachusetts and New York, separately.

Table A2: Characteristics of e-cigarette product use reported by a cross-sectional sample of youth and young adults in Massachusetts and New York State over two survey periods, unweighted sample size, weighted percentages and unweighted percentages

	**Massachusetts**					**New York State**				
	**Time 1**		**Time 2**		**p-value**	**Time 1**		**Time 2**		**p-value**
	(Feb 26, 2020-Apr 8, 2020)		Aug 26, 2020-Oct 20, 2020			(Feb 26, 2020-May 27, 2020)		(Aug 26, 2020-Jan 4, 2021)		
	n (wt. %)	unwt.%	n (wt. %)	unwt.%		n (wt. %)	unwt.%	n (wt. %)	unwt.%	
**<u>Sample Characteristics</u>**										
**Age**					0.985					0.987
**13-17**	352 (39.0%)	33.8	385 (38.7%)	38.9		1,014 (39.8%)	38.9	1,127 (39.8%)	41.3	
**18-20**	360 (27.5%)	34.5	268 (27.3%)	27.1		714 (26.4%)	27.4	750 (26.1%)	27.5	
**21-24**	330 (33.5%)	31.7	337 (34.0%)	34.0		877 (33.9%)	33.7	854 (34.1%)	31.3	
**Race/ethnicity**					0.998					0.995
**White NH**	676 (64.8%)	65.0	671 (64.9%)	68.1		1,492 (50.3%)	57.4	1,476 (50.2%)	54.4	
**Black NH**	72 (8.3%)	6.9	59 (8.2%)	6.0		319 (15.3%)	12.3	363 (15.6%)	13.4	
**Other NH, 2+ races**	129 (11.4%)	12.4	118 (11.6%)	12.0		318 (12.0%)	12.2	349 (12.0%)	12.9	
**Hispanic**	163 (15.5%)	15.7	138 (15.2%)	14.0		472 (22.5%)	18.1	523 (22.3%)	19.3	
**Gender**					0.825					0.778

**Male**	374 (50.0%)	35.9	512 (50.6%)	51.7		1,196 (50.3%)	45.9	1,409 (50.8%)	51.6	
**Female**	668 (50.0%)	64.1	478 (49.4%)	48.3		1,409 (49.7%)	54.1	1,322 (49.2%)	48.4	
**Financial Situation**					0.155					0.866
**Live comfortably**	461 (48.6%)	44.5	474 (50.6%)	48.0		1,264 (46.7%)	48.6	1,321 (47.5%)	48.5	
**Meet needs with a little left**	350 (31.3%)	33.8	321 (31.0%)	32.5		850 (32.9%)	32.7	872 (31.4%)	32.0	
**Just meet basic expenses**	197 (16.6%)	19.0	146 (13.1%)	14.8		405 (15.8%)	15.6	427 (16.3%)	15.7	
**Don't meet basic expenses**	29 (3.5%)	2.8	47 (5.3%)	4.8		80 (4.6%)	3.1	103 (4.8%)	3.8	
**E-cigarette use**										
**Ever**	462 (42.3%)	44.5	399 (39.6%)	40.5	0.317	1,130 (38.9%)	43.5	1,189 (40.6%)	43.7	0.329
**Current**	238 (22.7%)	22.8	194 (19.3%)	19.6	0.121	642 (20.1%)	24.7	733 (22.2%)	26.9	0.144
**Flavors used in past 30-days**										
**Tobacco**	55 (28.7%)	23.1	59 (32.4%)	30.4	0.523	121 (16.6%)	18.9	213 (26.2%)	29.1	0.001
**Menthol**	66 (30.5%)	27.7	39 (21.1%)	20.1	0.084	166 (26.4%)	25.9	202 (27.4%)	27.6	0.751
**Mint**	100 (40.8%)	42.0	37 (20.7%)	19.1	0.001	223 (36.7%)	34.8	200 (30.2%)	27.4	0.063
**Fruit**	114 (44.2%)	47.9	89 (50.8%)	45.9	0.265	308 (50.4%)	48.0	318 (47.1%)	43.5	0.363
**Other**	60 (25.0%)	25.2	58 (32.0%)	29.9	0.193	223 (32.4%)	34.8	260 (35.9%)	35.6	0.305
**Device type**					0.104					0.001
**Disposable**	71 (29.1%)	30.0	70 (38.2%)	37.4		180 (27.1%)	28.6	260 (38.2%)	36.8	

**Pod mod**	131 (56.0%)	55.3	81 (43.0%)	43.3		333 (48.7%)	52.9	330 (45.6%)	46.7	
**Tank/mod**	35 (14.9%)	14.8	36 (18.8%)	19.3		128 (24.2%)	18.4	116 (16.2%)	16.4	
**<u>Flavors by device type</u>**										
**Tobacco**					0.891					0.007
**Disposable**	15 (31.6%)	27.3	20 (36.5%)	35.7		32 (20.9%)	26.4	76 (32.6%)	36.0	
**Pod mod**	33 (53.2%)	60.0	27 (47.6%)	48.2		63 (46.1%)	52.1	106 (53.7%)	50.2	
**Tank/mod**	7 (15.2%)	12.7	9 (15.9%)	16.1		26 (33.1%)	21.5	29 (13.7%)	13.7	
**Menthol**					0.129					0.135
**Disposable**	14 (20.3%)	21.2	15 (43.0%)	38.5		32 (20.4%)	19.3	71 (31.7%)	35.3	
**Pod mod**	45 (68.5%)	68.2	21 (50.2%)	53.8		106 (55.4%)	63.9	104 (53.0%)	51.7	
**Tank/mod**	7 (11.3%)	10.6	3 (6.9%)	7.7		28 (24.1%)	16.9	26 (15.3%)	12.9	
**Mint**					0.934					0.64
**Disposable**	34 (34.9%)	34.0	9 (34.0%)	25.0		72 (32.5%)	32.3	67 (38.3%)	34.7	
**Pod mod**	61 (58.6%)	61.0	23 (56.9%)	63.9		114 (50.1%)	51.1	90 (44.9%)	46.6	
**Tank/mod**	5 (6.5%)	5.0	4 (9.0%)	11.1		37 (17.5%)	16.6	36 (16.8%)	18.7	
**Fruit**					0.062					0.004
**Disposable**	41 (33.3%)	36.0	34 (41.0%)	39.1		88 (30.2%)	28.6	130 (45.5%)	42.3	
**Pod mod**	53 (52.7%)	46.5	28 (33.1%)	32.2		148 (42.9%)	48.1	125 (39.1%)	40.7	
**Tank/mod**	20 (14.0%)	17.5	25 (26.0%)	28.7		72 (26.9%)	23.4	52 (15.3%)	16.9	
**Other flavors**					0.027					0.157
**Disposable**	16 (20.2%)	26.7	24 (47.2%)	42.9		59 (26.4%)	26.6	85 (37.5%)	34.3	

**Pod mod**	29 (58.0%)	48.3	23 (39.2%)	41.1		110 (48.8%)	49.5	113 (40.9%)	45.6	
**Tank/mod**	15 (21.8%)	25.0	9 (13.6%)	16.1		53 (24.8%)	23.9	50 (21.5%)	20.2	

© 2023 Schillo B. et al.
